# *In Vivo* Imaging of Glial Activation after Unilateral Labyrinthectomy in the Rat: A [^18^F]GE180-PET Study

**DOI:** 10.3389/fneur.2017.00665

**Published:** 2017-12-11

**Authors:** Andreas Zwergal, Lisa Günther, Matthias Brendel, Roswitha Beck, Simon Lindner, Guoming Xiong, Eva Eilles, Marcus Unterrainer, Nathalie Lisa Albert, Sandra Becker-Bense, Thomas Brandt, Sibylle Ziegler, Christian la Fougère, Marianne Dieterich, Peter Bartenstein

**Affiliations:** ^1^German Center for Vertigo and Balance Disorders, DSGZ, Ludwig-Maximilians-University, Munich, Germany; ^2^Department of Neurology, Ludwig-Maximilians-University, Munich, Germany; ^3^Department of Nuclear Medicine, Ludwig-Maximilians-University, Munich, Germany; ^4^Munich Cluster of Systems Neurology, SyNergy, Munich, Germany; ^5^Clinical Neurosciences, Ludwig-Maximilians-University, Munich, Germany; ^6^Department of Nuclear Medicine, Eberhard Karls University, Tübingen, Germany

**Keywords:** glial activation, vestibular compensation, small animal PET, translocator protein imaging, acute unilateral vestibulopathy

## Abstract

The functional relevance of reactive gliosis for recovery from acute unilateral vestibulopathy is unknown. In the present study, glial activation was visualized *in vivo* by [^18^F]GE180-PET in a rat model of unilateral labyrinthectomy (UL) and compared to behavioral vestibular compensation (VC) overtime. 14 Sprague-Dawley rats underwent a UL by transtympanic injection of bupivacaine/arsenilate, 14 rats a SHAM UL (injection of normal saline). Glial activation was depicted with [^18^F]GE180-PET and *ex vivo* autoradiography at baseline and 7, 15, 30 days after UL/SHAM UL. Postural asymmetry and nystagmus were registered at 1, 2, 3, 7, 15, 30 days after UL/SHAM UL. Signs of vestibular imbalance were found only after UL, which significantly decreased until days 15 and 30. In parallel, [^18^F]GE180-PET and *ex vivo* autoradiography depicted glial activation in the ipsilesional vestibular nerve and nucleus on days 7 and 15 after UL. Correlation analysis revealed a strong negative association of [^18^F]GE180 uptake in the ipsilesional vestibular nucleus on day 7 with the rate of postural recovery (*R* = −0.90, *p* < 0.001), suggesting that glial activation accelerates VC. In conclusion, glial activation takes place in the ipsilesional vestibular nerve and nucleus within the first 30 days after UL in the rat and can be visualized *in vivo* by [^18^F]GE180-PET.

## Introduction

Acute unilateral vestibulopathy (AUV) induces spontaneous nystagmus, head roll tilt, and falling to the lesion side (1, 2). Signs and symptoms recover over days to weeks due to central vestibular compensation (VC) (3). Therefore, VC is an interesting model for studying post-lesional plasticity in the adult intact brain (4). Several hypotheses have been proposed to explain VC. It is commonly accepted that VC is not a single process but involves multiple, synchronous, and synergistic adaptations in neuronal networks of various brain areas (5–9). The cellular and molecular mechanisms are still not completely understood. Microglial cells play an important role in brain plasticity following neuronal damage by modulating synaptic function, neurotransmission, and immune response (10, 11). Glial reactions in the ipsilesional vestibular nucleus (VN) after inner ear damage have been depicted by *in vitro* histochemistry (12–14). The functional relevance of microglia activation for vestibular nerve regeneration and central VC following inner ear damage is not known.

The 18 kDa translocator protein (TSPO), formerly known as peripheral-type benzodiazepine receptor, is a well-established marker for activated microglia (15, 16). It is predominantly located in the mitochondrial membrane and modulates the synthesis of neurosteroids (17). In the central nervous system (CNS), TSPO is expressed in microglia and in reactive astrocytes (16). In response to peripheral nerve injury, TSPO is upregulated in Schwann cells, macrophages, and neurons (18, 19). TSPO expression returns to resting levels only when nerve regeneration is completed, suggesting it plays an important role in nerve repair processes (20).

The aim of this study is to visualize vestibular nerve and cerebral glial activation after unilateral labyrinthectomy (UL) in rats *in vivo* and in correlation to behavior using PET with the novel TSPO ligand [^18^F]GE180 (21). The major advantage of this model is that it closely resembles the clinical picture of AUV, the most common cause of acute vertigo persisting for days. The present pilot study concentrates on the intermediate and late phases of postural compensation following a unilateral vestibular lesion.

## Materials and Methods

### Animals and Experimental Design

All animal experiments were approved by the Ethics committee of the University of Munich and the government of Upper Bavaria (number of license: 55.2-1-54-2532-93-16) and performed in accordance with the guidelines for the use of living animals in scientific studies and the EU and German Law for the protection of animals. Male Sprague-Dawley rats (mean 400 ± 20 g, age 3 months, Charles River Ltd., UK) were housed one animal per cage in a temperature- and humidity-controlled room with a 12-h light/dark cycle, with free access to food and water.

**Figure 1:**
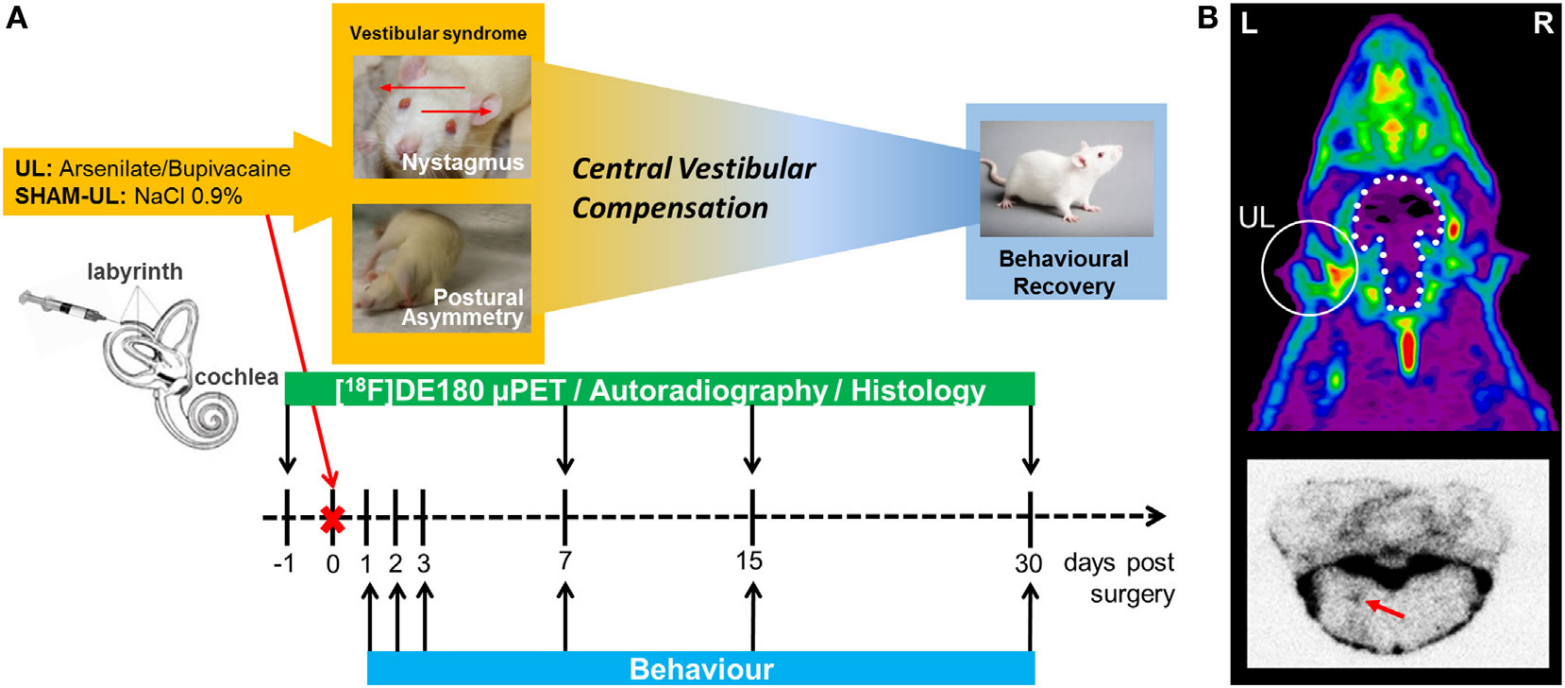
Study design for the rat unilateral labyrinthectomy (UL) model. **(A)** The timeline illustrates the different assessments and modalities in relation to the day of surgery. **(B)** The upper image represents a typical summed 60–90 min [^18^F]GE180 PET scan of the rat head in the axial direction. Localization of UL with corresponding translocator protein (TSPO) activation in the inner ear is indicated by a white circle. The white stippled line represents the position of the intact brain. The lower image represents a typical [^18^F]GE180 autoradiography slice of the brainstem, where elevated TSPO activity in the vestibular nucleus is highlighted with a red arrow. L, left; R, right.

The study was conducted in a partial longitudinal design (Table 1): from the 32 animals included in the experiment a baseline PET scan was performed prior to surgery in a randomized subgroup of six rats among which four were sacrificed for autoradiography and histology. The remaining rats were randomized into two groups of 14 animals to undergo either UL or SHAM-UL. PET was performed on day 7 (UL: *N* = 10, SHAM-UL: *N* = 6), day 15 (UL: *N* = 10, SHAM-UL: *N* = 5), and day 30 (UL: *N* = 4; SHAM-UL: *N* = 5). At each post-UL/SHAM-UL PET time point, four rats were sacrificed for *ex vivo* autoradiography and histology. Behavioral testing was performed in all available rats on days 1, 2, 3, 7 (UL/SHAM-UL: *N* = 14 each time point), day 15 (UL/SHAM-UL: *N* = 10), and day 30 (UL/SHAM-UL: *N* = 6) after surgery. Figure 1 illustrates the study design.

**Table 1:** Overview of the number of animals included in molecular imaging modalities and behavioral testing during the experiment.

Baseline (*N* = 32)	PET (*N*)			AR/HC (*N*)		
	
	6			4		

Surgery (*N* = 28)	UL (*N* = 14)			SHAM-UL (*N* = 14)		
	PET	AR/HC	Behavior	PET	AR/HC	Behavior
D1 (*N*)	–	–	14	–	–	14
D2 (*N*)	–	–	14	–	–	14
D3 (*N*)	–	–	14	–	–	14
D7 (*N*)	10	4	14	6	4	14
D15 (*N*)	10	4	10	5	4	10
D30 (*N*)	4	4	6	5	4	6

*PET, [^18^F]GE180-PET; AR, *ex vivo* autoradiography; HC, histochemistry; *N*, number of animals; UL, unilateral labyrinthectomy*.

### Unilateral Labyrinthectomy

Unilateral labyrinthectomy was performed as described in previous studies (22–25). Animals were anesthetized with 1.5% isoflurane delivered up to 1.2 l/min *via* a mask. For surgical analgesia, 1 mg/kg meloxicam was injected s.c. before and 3 days after surgery. After local anesthesia with 1% lidocaine hydrochloride, a left paramedian incision was made to expose the lamboidal ridge and the external ear canal. The external ear canal was opened just anterior to the exit point of the facial nerve. With a 26-gauge needle, the tympanic membrane was perforated caudally to the hammer shaft, and about 200 µl of a 20% bupivacaine solution was instilled into the tympanic cavity. After about 1 min, the bupivacaine solution was aspirated and instilled slowly again. This process was repeated three times. After the local anesthetic was instilled, the same procedure was performed to instill 200 µl of a 10% solution of p-arsanilic acid, which irreversibly desensitized the primary sensory cells of the inner ear (26). In the control group, 200 µl of 0.9% saline was instilled three times into the tympanic cavity [in the following the process is called sham unilateral labyrinthectomy (SHAM-UL)]. After the last thorough aspiration, the wound was closed by skin suture and 1 mg/kg marbofloxacine was injected s.c. for 3 days for preventive antibiosis.

### Criteria for Exclusion

Animals were excluded from the study if the following symptoms were observed:

– loss of more than 20% of the pre-treatment body weight,
– ulcer of the cornea, which could occur due to an inadvertent lesion of the facial nerve,
– bleeding from the tympanic cavity, which could prevent the diffusion of bupivacaine or p-arsanilic acid into the inner ear,
– abnormality in behavioral scoring, e.g., convulsions, paresis, or hemiataxia.

### Behavioral Testing after UL/SHAM-UL

The behavioral symptoms of vestibular imbalance nystagmus and postural asymmetry were scored after unilateral vestibular ablation (27). Each component was given a maximum score of 10:

– *Postural deficits* were scored as follows: spontaneous barrel rolling—10 points; barrel rolling evoked by a light touch or air-puff—9 points; recumbent position on lesion side without leg support—8 points; some ipsilesional leg support—7 points; moving around on one side or using ipsilesional legs for recumbent support—6 points; moving around with bilateral leg support—5 points; moving around with occasional falls to the ipsilesional side—4 points; moving around leaning toward the ipsilesional side—3 points; hardly noticeable asymmetry—2 points; postural asymmetry only noticeable when picked up—1 point;
– *Nystagmus* was observed visually. Intensity of spontaneous nystagmus was scored with 6–10 points, with 1 point for every 60 beats per minute (bpm). If spontaneous nystagmus was absent at rest, the animal was touched slightly. If this evoked nystagmus, a score of 1–5 points was given, with 1 point for every 60 bpm.

The estimated function of VC was calculated by using serial postural instability scores starting on day 2 post surgery and ending at the final observation point (≥day 7) of individual rats. Day 1 was excluded to avoid any bias from delayed anesthesia effects. Thus, a minimum of three observation points were considered for each rat. For each rat, the average change per day was computed by the slope of the linear function.

### [^18^F]GE180 Synthesis

Automated production of GE180 was performed on a FASTlab^TM^ (GE Healthcare) synthesizer with single-use disposable cassettes (28). The pre-filled precursor vial was assembled on the cassette, and the cassette was mounted on the synthesizer according to the set-up instructions. The FASTlab^TM^ control software prompts were followed to run the cassette test and to start the synthesis. No carrier added ^18^F-fluoride was produced *via* ^18^O(p, n)^18^F reaction by proton irradiation of ^18^O-enriched water and delivered to the ^18^F incoming reservoir. The fully automated manufacturing process consists of the following steps: trapping of ^18^F-fluoride on a QMA cartridge, elution using Kryptofix^®^222, potassium hydrogen carbonate, water and acetonitrile, azeotropic drying of ^18^F-fluoride at 120°C for 9 min, labeling of the precursor in MeCN at 100°C for 6 min, dilution of the crude product with water, tC18 cartridge-based purification by using 20 mL 40% (v/v) Ethanol and 11.5 mL 35% (v/v) Ethanol, elution of the product with 3.5 mL 55% (v/v) Ethanol and final formulation with phosphate buffer. Synthesis time was 43 min, radiochemical yield (non-decay corrected) was 34 ± 9% (*n* = 18) with radiochemical purity ≥98%, and specific activity was 1810 ± 616 GBq/μmol (end of synthesis).

### PET Imaging

PET imaging was performed at the preclinical research division of the Department of Nuclear Medicine at the University of Munich. For the animal studies, anesthesia was induced with isoflurane (as described above), and a cannula was placed in a tail vein. Animals were positioned in the Siemens Inveon P120 PET scanner (Siemens Medical Solutions, Munich, Germany) and were kept warm with a heating pad. To prevent head movements, the head position was fixed using a custom-made head holder. A bolus of 50 MBq [^18^F]GE180 in 0.5 ml saline was injected and a 90-min dynamic emission recording was initiated followed by a 15-min transmission scan using a rotating [^57^Co] point source. After recovering from anesthesia, the rats were returned to their home cages.

### Image Processing and Statistical PET Analysis

Emission recordings were corrected for random, coincidences, normalized, and reconstructed with iterative reconstruction employing the Ordered Subsets Expectation Maximization (OSEM-3D) algorithm, which includes scatter and attenuation correction (Siemens Medical Solutions Munich, Germany) and results in a final 128 × 128 × 159 matrix. For attenuation correction, the corresponding transmission measurements at the end of the emission scan were used. The voxel dimensions of the reconstructed images were 0.78 mm × 0.78 mm × 0.80 mm. Further data processing was performed by PMOD (V3.5, PMOD Technologies Ltd.). The 60–90 min time frame was selected for further analyses as this time frame proved to be suitable in a recent in-house investigation in rodents (29). Static datasets (60–90 min) were co-registered to a high-resolution rat cryo-atlas by a manual rigid-body transformation (TX_rigid_) using the PMOD fusion tool (V3.5, PMOD Technologies Ltd.), after blinding the rat identity to the reader. In the second step, a reader-independent fine co-registration to a tracer-specific template was performed (30). The template was generated by averaging all PET scans. Then, the initial manual PET-to-atlas fusion images were normalized by non-linear brain normalization (TX_BN_) to the tracer-specific template using the PMOD brain normalization tool (equal modality; smoothing by 0.8 mm; nonlinear warping; 16 iterations; frequency cutoff 3; regularization 1.0; no thresholding). The concatenation of TX_rigid_ and TX_BN_ was then applied to PET datasets in the native space to obtain optimal resampling with a minimum of interpolation. [^18^F]GE180 data were scaled by the global cerebral mean. Voxel-wise analyses were conducted by custom-made toolboxes, implemented in statistical parametric mapping software SPM5 (Wellcome Department of Cognitive Neurology, London). Images were used after pre-processing. For each timepoint after surgery groups of UL and SHAM-UL, rats were compared by an unpaired Student’s *t*-test. Additionally, SHAM-UL rats of all time points were compared against the baseline to test for purely surgery-related effects. A statistical threshold of *p* < 0.01 (uncorrected for multiple comparisons) and a voxel-threshold of 50 were defined.

Volume-of-interest (VOI) based analyses were performed in brain volumes deriving from voxel-wise analyses. Standardized uptake value ratios including global mean scaling (SUVR_GLM_) for the ipsilesional VN and nerve were extracted from individual animals and used for further group comparisons and correlation analyses.

### *Ex Vivo* Autoradiography and Histology

After completion of the attenuation scans (110 min after injection of [^18^F]GE180) four rats per group (UL/SHAM-UL) and time point (days 7, 15, 30) after UL/SHAM-UL were sacrificed by intracardiac injection of 0.4 mg/kg xylazinhydrochloride (Rompun^®^) in deep isoflurane narcosis. Brains were resected and immediately frozen at −80 °C, followed by thermal equilibration at −20 °C for 10 min in a Leica CM 1510-1 Cryostat (Leica Microsystems, Nussloch Germany). Then the brains were cut in 20-μm-thick transverse slices, which were mounted on microscope slides (Menzel GmBH, super frost, Germany) and placed on Fujifilm BAS cassette2 2025 imaging plates. The plates were exposed for 24 h and then scanned at standard resolution, 25 µm, with the Raytest equipment (CR-Reader Software, v.1.4.1., Dürr Medical, Germany). Regions of interest (ROIs) were drawn on the ipsi- und contralesional vestibular and facial nucleus (FN). The quantitative luminescence value (QL) for each ROI was determined using Advanced Image Data Analyzer (AIDA, v.4.5), relative to the reference tissue. The reference region was determined as the background QL measured in the midline pons individually for every brain section. For anatomic coregistration, the transverse brain slices, which were used for autoradiography, underwent a subsequent Nissl-staining process using established protocols (31).

### Statistics Analysis

IBM SPSS Statistics (version 23.0; SPSS, Chicago, IL, USA) was used for all statistical tests. Normal distribution was tested by the Kolmogorov–Smirnov test. Statistical group comparison for behavioral scoring was performed by the non-parametric Kruskal–Wallis *H* test and *post hoc* testing with Bonferroni correction to analyze significant differences between time points after UL. PET SUVR_GLM_ values were compared between UL and SHAM-UL groups by an unpaired Student’s *t*-test. Autoradiography QL/pixel values of vestibular and facial nuclei were compared between ipsi- and contralateral lesion side by a paired Student’s *t*-test and between UL and SHAM-UL groups by an unpaired Student’s *t*-test. Pearson’s coefficient of correlation was calculated between regional TSPO activity (SUVR_GLM_) and changes in postural instability per day. A significance level of *p* < 0.05 was set for rejection of the null hypothesis.

## Results

### Behavioral Compensation after UL

**Figure 2:**
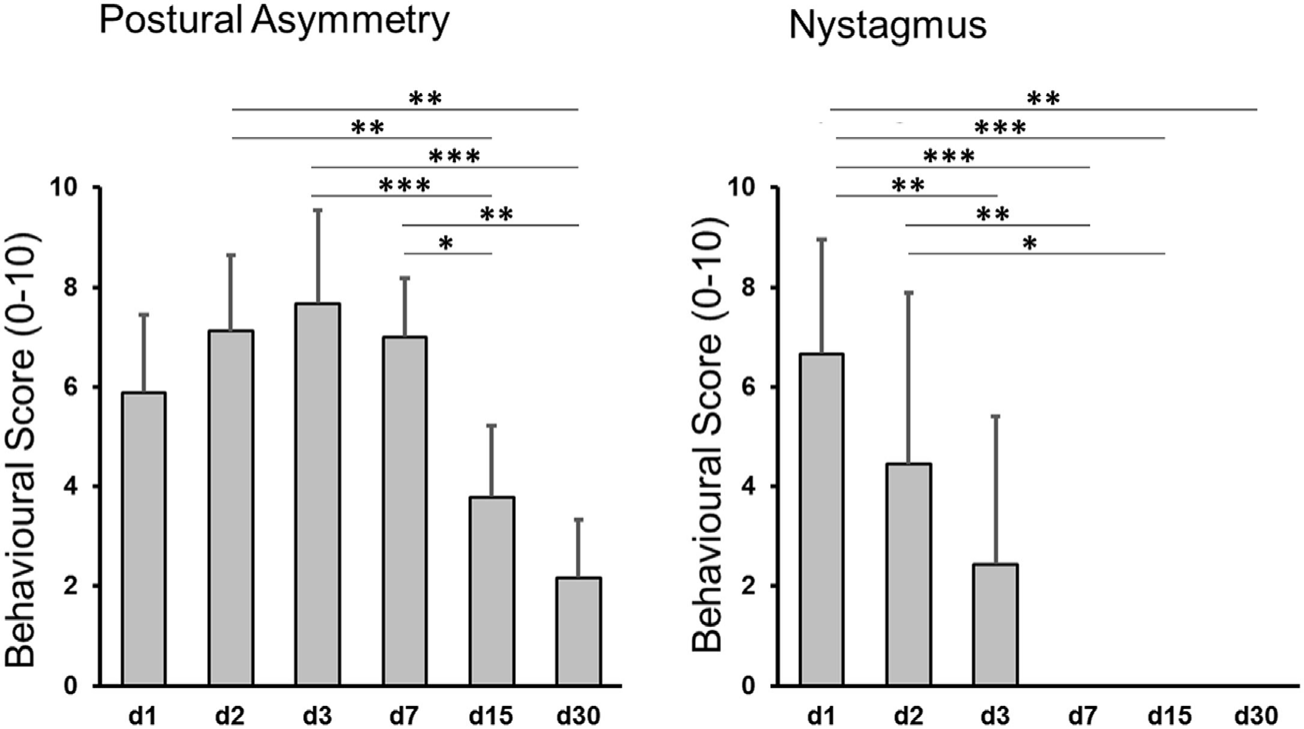
Behavioral scores after unilateral labyrinthectomy (UL). Postural imbalance scores decreased significantly until day 30 post-UL (left side), nystagmus until day 7 post-UL (right side). Values are depicted as mean ± SD. Significant differences between time points are depicted based on *post hoc* analysis of the Kruskal–Wallis *H* test: *p < 0.05, **p < 0.005, ***p < 0.0005.

After UL all animals showed severe vestibular imbalance including nystagmus, barrel rolling, circular walking, and postural instability, whereas no signs of vestibular dysfunction were observed after SHAM surgery. These data are comparable with results of previous studies using the same UL model (23, 25). In the UL group, postural asymmetry significantly improved until day 30 (mean postural scores 7.4 ± 2.0 on day 3 versus 4.2 ± 1.3 on day 15, and versus 2.1 ± 1.2 on day 30). The Kruskal–Wallis *H* test showed that there was a significant difference in postural scores between days after UL, χ^2^(5) = 39.258, *p* < 0.0005. *Post hoc* tests with Bonferroni correction revealed significant differences between days 15 and 2 (*z* = 3.649, *p* = 0.004), day 3 (*z* = 4.440, *p* < 0.0005), and day 7 (*z* = 3.226, *p* = 0.019), as well as days 30 and 2 (*z* = 4.127, *p* = 0.001), day 3 (*z* = 4.789, *p* < 0.0005), and day 7 (*z* = 3.774, *p* = 0.002) (Figure 2). Spontaneous nystagmus ceased until day 7 (mean nystagmus scores 6.0 ± 2.3 on day 1 versus 1.9 ± 3.2 on day 3). For nystagmus the Kruskal–Wallis *H* test showed a significant difference between days after UL. *Post hoc* tests indicated significant differences between days 3 and 1 (*z* = 3.792, *p* = 0.002), days 7 and 1 (*z* = 5.777, *p* < 0.0005), days 15 and 1 (*z* = 4.953, *p* < 0.0005), days 30 and 1 (*z* = 4.114, *p* = 0.001), as well as days 7 and 2 (*z* = 3.843, *p* = 0.002) and days 15 and 2 (*z* = 3.295, *p* = 0.015) (Figure 2).

### [^18^F]GE180-PET Analysis after UL versus SHAM Surgery

[^18^F]GE180-PET scans were performed at baseline and on days 7, 15, and 30 after UL or SHAM-UL surgery to investigate distribution and kinetics of TSPO activity *in vivo* during the later course of behavioral compensation. Successfully imaged numbers of rats are reported in Table 1. TSPO polymorphisms were excluded by genetic testing.

**Figure 3:**
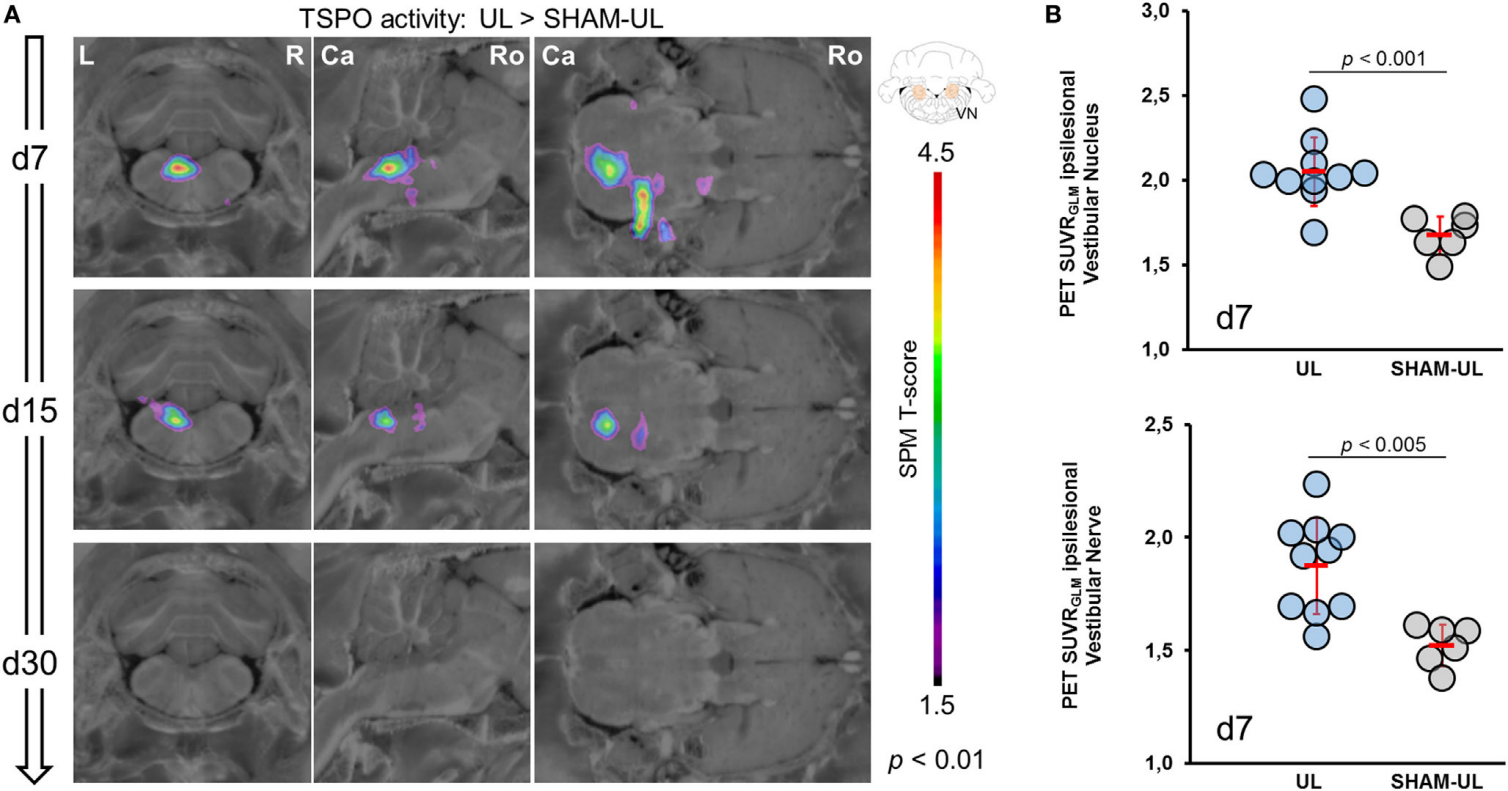
Voxel-wise and VOI-based comparison of [^18^F]GE180-PET images between UL and SHAM-UL groups. **(A)** In the UL group, translocator protein (TSPO) activity was increased in the ipsilesional VN and nerve on day 7 after surgery (UL: *N* = 10 versus SHAM-UL: *N* = 6 rats). Both regions indicated still elevated TSPO activity at a lower degree on day 15 (UL: *N* = 10 versus SHAM UL: *N* = 5 rats). No significant differences in [^18^F]GE180 accumulation between both groups were found on day 30 (UL: *N* = 4 versus SHAM-UL: *N* = 5 rats). **(B)** VOI-based values of single UL rats (*N* = 10) on day 7 indicate elevated but heterogeneous TSPO activation in the vestibular nerve and nucleus when compared to SHAM-UL (*N* = 6). UL, unilateral labyrinthectomy; R, right; L, left; Ca, caudal; Ro, rostral; VN, vestibular nucleus; SUVR_GLM_, standardized uptake value ratios including global mean scaling; VOI, volume-of-interest.

SPM-based analysis of group data revealed a strongly elevated [^18^F]GE180 uptake in the ipsilesional VN and nerve at day 7 post-UL when compared to SHAM-UL (*p* < 0.01) (Figure 3A). [^18^F]GE180 uptake remained significantly increased in the VN and nerve on the lesion side on day 15 post-UL (*p* < 0.001). On day 30, [^18^F]GE180 accumulation did not indicate a statistical difference in any brain region when comparing UL and SHAM-UL rats. The same [^18^F]GE180 uptake pattern was found when values for days 7, 15, and 30 post-UL were compared to baseline. [^18^F]GE180 uptake in the SHAM surgery group did not differ from baseline at any time point (data not shown).

Volume-of-interest-based analysis in the ipsilesional VN indicated a significantly increased [^18^F]GE180 SUVR_GLM_ after UL as compared to SHAM surgery on day 7 [2.05 ± 0.22 versus 1.68 ± 0.11, *t* (14) = 4.17, *p* = 0.0009, Figure 3B] and on day 15 [1.99 ± 0.12 versus 1.79 ± 0.17, *t* (13) = 2.48, *p* = 0.0275], whereas [^18^F]GE180 SUVR_GLM_ was nearly normalized on day 30 [1.85 ± 0.17 versus 1.78 ± 0.17, *t* (7) = 0.41, *p* = 0.6950]. [^18^F]GE180 uptake in the ipsilesional vestibular nerve was likewise significantly increased on day 7 in the UL group as compared to the SHAM surgery group [1.88 ± 0.21 versus 1.52 ± 0.09, *t* (14) = 3.82, *p* = 0.0019, Figure 3B]. In the same comparison, TSPO activity in the nerve was still elevated on day 15 [1.79 ± 0.24 versus 1.45 ± 0.13, *t* (13) = 2.66, *p* = 0.0195], and indicated a trend to prolonged elevation on day 30 [1.71 ± 0.12 versus 1.41 ± 0.17, *t* (7) = 1.83, *p* = 0.1095]. Baseline values prior to surgery were in the range of SHAM-UL for the VN (1.71 ± 0.08) and the vestibular nerve (1.47 ± 0.18).

### Correlation of TSPO Activity and VC

**Figure 4:**
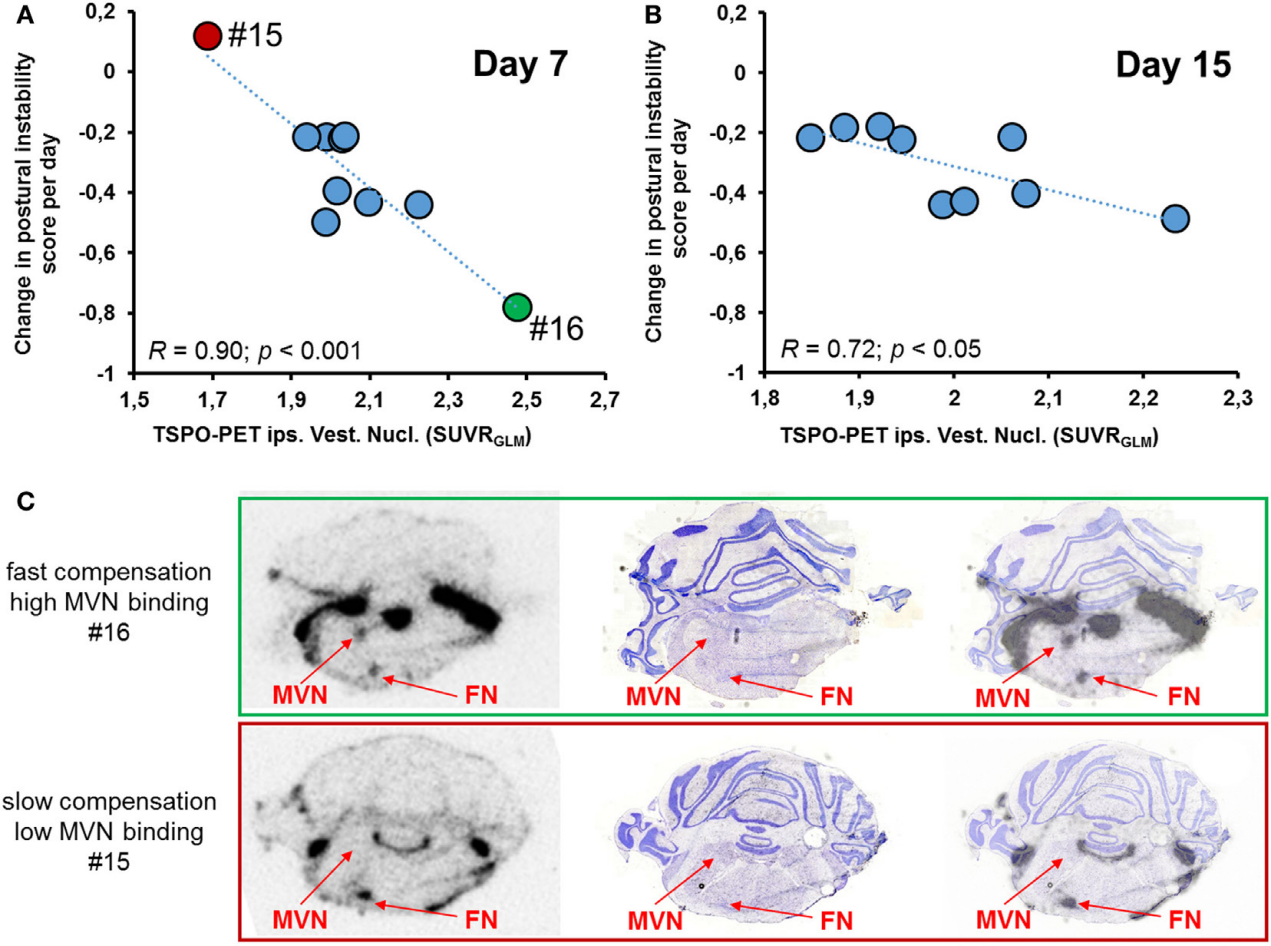
Correlative analysis of SUV [^18^F]GE180 uptake in the ipsilesional vestibular nucleus (VN) and behavioral scores. **(A)** [^18^F]GE180 uptake on day 7 post-unilateral labyrinthectomy (UL) correlated negatively with the daily changes in postural asymmetry scores of UL rats (*N* = 10). **(B)** The negative correlation between translocator protein (TSPO) activation in the ipsilesional VN and the degree of compensation was still present for [^18^F]GE180 uptake on day 15 post-UL (*N* = 9 rats). **(C)** Exemplary autoradiography at the level of the VN and facial nucleus (FN) (including anatomical verification by histology) from a rat with very fast vestibular compensation (VC) until death on day 7 (green, upper image, high [^18^F]GE180 uptake) and another slice from a rat with very slow VC until death on the same day (red, lower image, low [^18^F]GE180 uptake).

Correlation analysis of [^18^F]GE180-PET on day 7 was done with parameters of postural asymmetry only as nystagmus remission was completed at that time. TSPO activity in the ipsilateral VN on day 7 correlated highly with the slope of compensation (assessed by postural asymmetry) on the single rat level (*r* = −0.90; *p* < 0.001; Figure 4A). This association was present at a lower level when correlating levels of TSPO activity in the ipsilateral VN on day 15 with the slope of compensation (*r* = 0.72; *p* < 0.05; Figure 4B), whereas no significant correlation was found for day 30. Behavioral scores did not correlate with TSPO activity in the ipsilateral vestibular nerve eventually. Exemplary *ex vivo* autoradiography findings on day 7 of two rats with highly different compensation slopes confirmed the *in vivo* findings (Figure 4C).

### *Ex Vivo* [^18^F]GE180 Autoradiography and Histology

**Figure 5:**
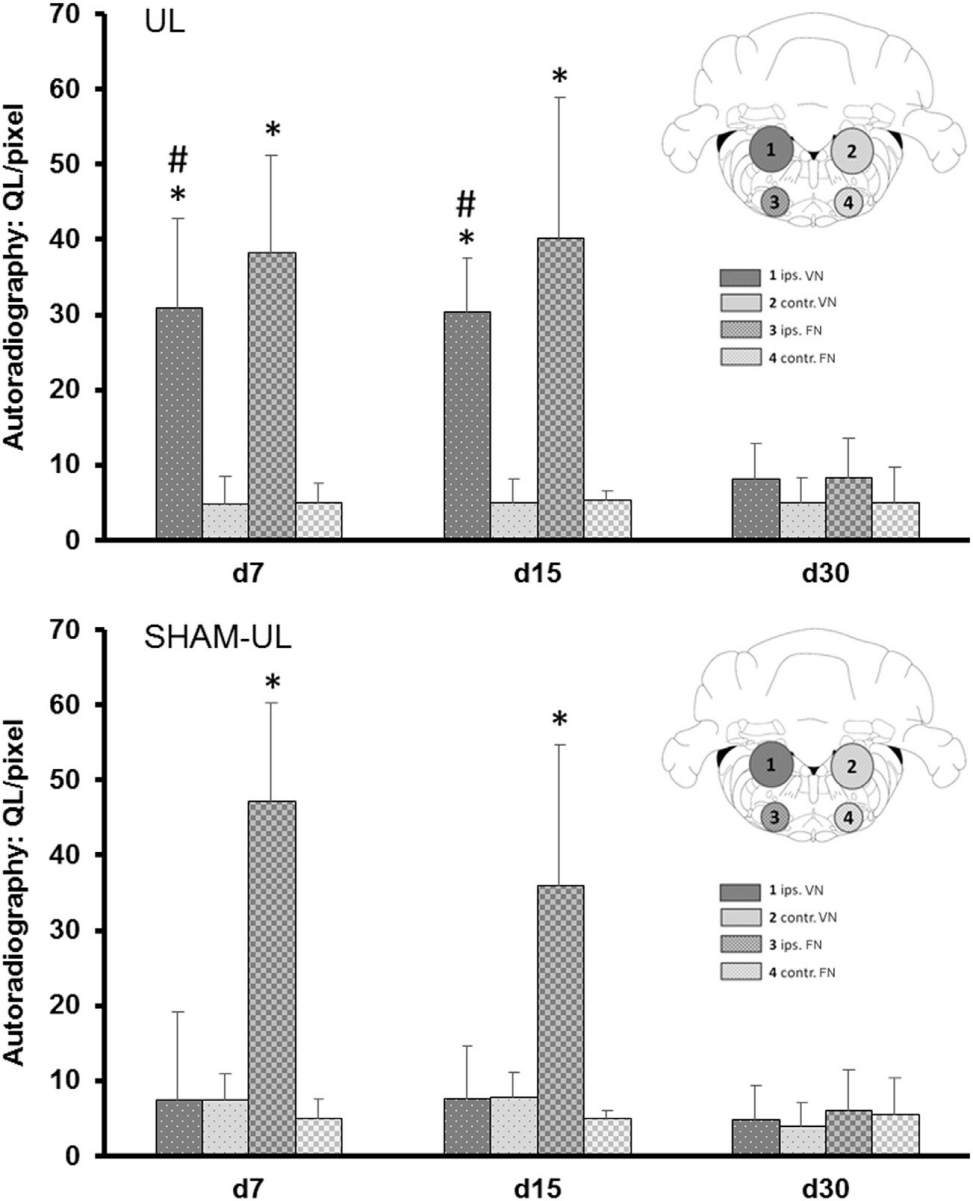
Quantitative ROI-based analysis of *ex vivo* [^18^F]GE180 autoradiography after UL and SHAM-UL. Significant increases of [^18^F]GE180 uptake were found in the ipsilesional VN on days 7 and 15 after UL, when compared to contralateral, but no significant differences were observed in the VN of SHAM-UL. [^18^F]GE180 uptake was consistently elevated in the ipsilateral FN on days 7 and 15 after UL and SHAM-UL, when compared to contralateral (UL/SHAM-UL: *N* = 4 rats, respectively, per time point). UL, unilateral labyrinthectomy; ips, ipilesional; contr, contralesional; VN, vestibular nucleus; FN, facial nucleus; ROI, region of interest. *p < 0.05 ipsilateral versus contralateral; ^#^*p* < 0.05 UL versus SHAM-UL.

The pattern of [^18^F]GE180 binding determined by autoradiography *ex vivo* qualitatively resembled the binding differences observed in the PET scans. Further quantitative analyses were performed in histologically confirmed ROIs (i.e., ipsilesional VN, contralesional VN, ipsilesional FN, contralesional FN). The binding of [^18^F]GE180 in UL rats was significantly increased sixfold in the ipsilesional VN at day 7 and sixfold at day 15 as compared to the contralesional VN [*t* (6) = 3.16/3.34, both *p* < 0.05] (Figure 5). In SHAM-UL, there was no significant asymmetry of [^18^F]GE180 binding in the vestibular nuclei. [^18^F]GE180 binding appeared increased by seven- to ninefold in the ipsilesional FN on days 7 and 15 in UL and SHAM-UL groups [*t* (6) = 3.66/2.74, both *p* < 0.05], potentially indicating irritation of the facial nerve due to middle ear injection.

## Discussion

To the best of our knowledge, this is the first study to show glial activation *in vivo* during VC in a rat model of a unilateral inner ear lesion using a novel TSPO ligand μPET approach. The major findings of the study are the following: (1) toxic inner ear damage induces a glial activation in the vestibular nerve and nucleus on the lesion side within the first 30 days. (2) Glial activation parallels the course of late postural compensation.

### [^18^F]GE180 PET Imaging of Glial Activation during VC and Recovery

The TSPO is a mitochondrial transporter, which has a relatively low expression in the healthy CNS. However, various pathologies can cause significant upregulation of TSPO expression (16). In the past, the TSPO radiotracer ligand [^11^C]PK11195 was successfully applied to depict glial activation in various CNS diseases like multiple sclerosis, Huntington’s disease, amyotrophic lateral sclerosis, Alzheimer’s disease, traumatic brain injury, and ischemic stroke (32–35). A recent [^11^C]PK11195-PET study in the rat showed spinal glial activation after partial sciatic nerve ligation with peaking kinetics at 7–14 days post-lesion (36).

The new second-generation TSPO ligand [^18^F]GE180 has so far not been applied to visualize glial activation in a model of peripheral nerve or sensory organ damage (21). We here report [^18^F]GE180 uptake in the vestibular nerve and nucleus with on days 7 and 15 post-chemical UL in the rat. Exact localization of glial activation was validated by *ex vivo* autoradiography and histology. The dynamics and localization of microglial activation depicted by [^18^F]GE180-PET and autoradiography nicely confirm previous data from *in vitro* studies (13, 14). A methodological advantage of the chemical UL model, which is a sensory end-organ lesion model, is that cerebral networks remain completely intact. Confounding effects of a blood–brain barrier disturbance, cerebral perfusion changes, or systemic inflammation on [^18^F]GE180 uptake can therefore be ruled out. A further advantage of the present study in the UL model is that behavioral changes of vestibular imbalance can be easily recorded, quantified, and correlated to the [^18^F]GE180 uptake, which allows further insights into the functional relevance of glial activation for behavioral compensation after inner ear damage *in vivo*.

### The Functional Role of Microglial Activation in VC

Past *in vitro* studies described microglial activations within the VN following inner ear damage by means of mRNA analysis and immunostaining (12–14). However, the mechanisms inducing central microglial activation after a peripheral vestibulo-cochlear damage and the functional relevance of microglial activation for VC remain unclear. It has been hypothesized that not the decrease in neuronal activity *per se* but rather trans-neuronal changes of inflammatory cytokines may induce microglial activation in the VN (14). Comparative analysis of behavioral and [^18^F]GE180 kinetics in our study suggest that microglial activation in the VN initially parallels the occurrence of vestibular imbalance measured in postural asymmetry scores (Figures 2 and 3). Therefore, it is most likely that the vestibular damage trans-neuronally drives glial activation along the vestibular nerve and nucleus. Potential mechanisms may be the local modification of neuropeptide expressions, cytokines, and growth factors triggered by impending vestibular nerve degeneration (19, 37, 38). Interestingly, no other brain region besides the ipsilesional VN showed [^18^F]GE180 uptake.

Our data furthermore suggest that this glial activation in the VN may be beneficial for further VC and recovery. Higher [^18^F]GE180 binding in the VN, but not vestibular nerve, especially on day 7 correlated with faster compensation of postural imbalance (Figure 4). It is well-accepted that structural plasticity in the VN and commissural system is a central mechanism of VC (3, 9). Glial reaction may counteract a degeneration of second-order vestibular neurons and improve recovery of their neuronal resting activity and may change balance of excitatory and inhibitory neurotransmission in the VN (14). In analogy to our data, a potentially beneficial role of glial activation has been described in other peripheral nerve disorders (19, 39). Microglial activation after peripheral nerve lesion persists as long as functional nerve damage is present and ceases only upon regeneration (20). Microglial activation by TSPO ligands may potentially promote functional recovery in experimental peripheral nerve lesions (16, 40).

### Clinical Relevance of Microglial Activation in AUV

In clinical practice, AUV is the most frequent reason for presentation of acute vertigo with postural imbalance persisting for days to weeks (41). However, the pathophysiology of this clinical disorder is not known. Inflammatory changes along the vestibular nerve have been hypothesized (41, 42).

What can a rat model of UL contribute to this discussion? It definitely shows that even in case of toxic damage to the vestibular and cochlear hair cells of the inner ear, there is a secondary inflammatory response *via* microglial activation along the vestibular nerve leading to the VN. It could well be that secondary degeneration of the Scarpa’s ganglia and the vestibular nerve plays a critical role in glial activation during the later stages after UL (23). However, the present study shows that any damage of the peripheral vestibular system seems to induce a reactive neuroinflammation. The data of the current study suggest that conceptually it seems important to think about AUV as a disease with various etiologies, which results in a common pathophysiological neuroinflammatory response of the first- and second-order vestibular neurons.

### Limitations

The current pilot study aimed to establish *in vivo* imaging of glial activation by means of [^18^F]GE180-PET in the rat model of chemical UL. The imaging time points were guided to the later phase of VC after day 7. Thereby early mechanismens of VC, happening during the first days after UL were neglected. As concerns comparative interpretation of PET and behavioral data, the correlation of TSPO activity with the slope of postural improvement does not equal causation. Therefore, the role of glial activation in VC needs further investigation, in order to determine if it is beneficial or not. A partial longitudinal study design was chosen to be able to compare and validate TSPO activity in PET with the *ex vivo* autoradiography signal in a subset of rats. However, this design has the disadvantage that the number of animals drops during the experiment. This could cause problems for the statistical assumptions, such as homogeneity of variance. Differences in numbers of PET scans on day 30 versus days 15 or 7 may contribute to the lack of significance in SPM between UL and SHAM-UL at that time point. However, *ex vivo* autoradiography and VOI-based *in vivo* analyses likewise resulted in only minor differences on day 30. In summary, differences in glial activity between UL and SHAM-UL during the very late phase of VC after UL should be negligible.

## Conclusion

The present rat [^18^F]GE180-PET study shows *in vivo* evidence that microglial activation appears in the vestibular nerve and nucleus following inner ear damage. The model of chemical UL has the unique advantage that the vestibular nerve and central networks remain structurally intact. This allows investigation of microglial reactions without any contamination due to structural cerebral damage. The pattern of glial activation indicates that a reactive neuroinflammation of the vestibular nerve and nucleus evolves after peripheral deafferentation, which may be beneficial for functional recovery.

## Ethics Statement

All animal experiments were approved by the Ethics committee of the University of Munich and the government of Upper Bavaria (number of license: 55.2-1-54-2532-93-16) and performed in accordance with the guidelines for the use of living animals in scientific studies and the EU and German Law for the protection of animals.

## Author Contributions

AZ, NA, and PB contributed to planning of the study, analysis and interpretation of data, and writing of the manuscript. LG, RB, and EE contributed to planning of the study, execution of experiments, analysis of data, and writing of the manuscript. MB, GX, MU, and SB-B contributed to analysis of data and writing of the manuscript. SL contributed to execution of experiments and writing of the manuscript. TB, SZ, CF, and MD contributed to planning of the study and writing of the manuscript.

## Conflict of Interest Statement

The authors declare that the research was conducted in the absence of any commercial or financial relationships that could be construed as a potential conflict of interest.
